# Carcinoma-associated fibroblasts affect sensitivity to oxaliplatin and 5FU in colorectal cancer cells

**DOI:** 10.18632/oncotarget.11121

**Published:** 2016-08-08

**Authors:** Samuel Gonçalves-Ribeiro, Natalia Guillen Díaz-Maroto, Mireia Berdiel-Acer, Antonio Soriano, Jordi Guardiola, Mercedes Martínez-Villacampa, Ramon Salazar, Gabriel Capellà, Alberto Villanueva, Eva Martínez-Balibrea, David G. Molleví

**Affiliations:** ^1^ Program Against Cancer Therapeutic Resistance, Catalan Institute of Oncology, IDIBELL, L'Hospitalet de Llobregat, Catalonia, Spain; ^2^ Gastroenterology Department, Endoscopy Unit, Hospital Universitari de Bellvitge, IDIBELL, L'Hospitalet de Llobregat, Catalonia, Spain; ^3^ Medical Oncology Department, Catalan Institute of Oncology, IDIBELL, L'Hospitalet de Llobregat, Catalonia, Spain; ^4^ Hereditary Cancer Program, Catalan Institute of Oncology, IDIBELL, L'Hospitalet de Llobregat, Catalonia, Spain; ^5^ Program Against Cancer Therapeutic Resistance, Catalan Institute of Oncology, IGTP, Badalona, Catalonia, Spain

**Keywords:** carcinoma-associated fibroblasts, microenvironment-mediated drug resistance, colorectal cancer, resistance, chemotherapy

## Abstract

The importance of tumor microenvironment (TME) as a relevant contributor to cancer progression and its role in the development of *de novo* resistance to targeted therapies has become increasingly apparent. However, the mechanisms of microenvironment-mediated drug resistance for nonspecific conventional chemotherapeutic agents, such as platinum compounds or antimetabolites, are still unclear.

Here we describe a mechanism induced by soluble factors released by carcinoma-associated fibroblasts (CAFs) that induce the translocation of AKT, Survivin and P38 to the nucleus of tumor cells. These changes are guided to ensure DNA repair and the correct entrance and exit from mitosis in the presence of chemotherapy. We used conditioned media (CM) from normal-colonic fibroblasts and paired CAFs to assess dose response curves of oxaliplatin and 5-fluorouracil, separately or combined, compared with standard culture medium. We also evaluated a colony-forming assay and cell death to demonstrate the protective role of CAF-CM. Immunofluorescence confirmed the translocation of AKT, P38 and Survivin to the nucleus induced by CAF-soluble factors. We also have shown that STAT3 or P38 inhibition provides a promising strategy for overcoming microenvironment-mediated resistance. Conversely, pharmacologic AKT inhibition induces an antagonistic effect that relieves a cMET and STAT3-mediated compensatory feedback that might explain the failure of AKT inhibitors in the clinic so far.

## INTRODUCTION

Colorectal cancer (CRC) is the fourth most prevalent cancer and the leading cause of cancer mortality worldwide [1], but its early diagnosis makes the disease one of the most curable cancers [2], at least in developed countries. Nevertheless, one of the main obstacles that patients have to deal with over the course of the disease is resistance to treatments.

Research into therapies against specific targets or signaling pathways is one of the pillars of current cancer research, although most tumors are still treated with conventional cytotoxic therapies. Drug resistance remains the main obstacle to the success of cytotoxic therapies [3]. As with many aspects of the tumorigenic process, most research on drug resistance has focused on the acquired resistance of malignant cells, which is basically limited to the reduction of an initial tumor burden, and so fails to eradicate a sufficient number of cancer cells to prevent clinical recurrence. Despite the extensive use of chemotherapy, the specific mechanisms causing tumor regression or recurrence after treatment are poorly known.

Environment-mediated drug resistance is a form of *de novo* resistance in which tumor cells are transiently protected from drugs [4]. The elevated serum levels of several cytokines secreted mainly by CAFs, such as IL-8, IL-1β, VEGF, TNFα, IL-17 and IL-6, have a prognostic value and are also implicated in tumor aggressiveness and poor response to therapy [5]. Signaling events triggered by such stromal cytokines and growth factors may be involved in *de novo* resistance, contributing to the failure to eliminate minimal residual disease, resulting, after strong selective pressure of therapy, in the recruitment of cancerous cells with acquired-resistance phenotypes [6, 7]. This protective effect is not universal across tumor types and drugs [8]. The effect of the microenvironment on resistance to targeted therapies is easier to understand conceptually, since different soluble factors might activate signaling events converging in the same pathway downstream of the targeted molecule/receptor. However, the mechanisms of microenvironment-mediated drug resistance for nonspecific and pleiotropic conventional chemotherapeutic agents, such as platinum compounds and antimetabolites, are still unclear.

Here we explore how CAF-soluble factors contribute to CRC chemoresistance in the presence of antimetabolites and DNA-damaging agents, like 5-fluorouracil (5FU), oxaliplatin (L-OHP). To this end, we decided to investigate multiple signaling pathways that may be involved in mediating resistance and that might offer a useful approach to identifying and describing some cellular and molecular alterations in the CRC chemoresistance process. We also examined how colorectal cancer cells may be sensitized to chemotherapy, in order to overcome the chemoresistance induced by CAFs.

## RESULTS

### Altered chemosensitivity of colorectal cancer cells after continuous exposure to chemotherapy in the presence of conditioned media from CAFs

**Figure 1:**
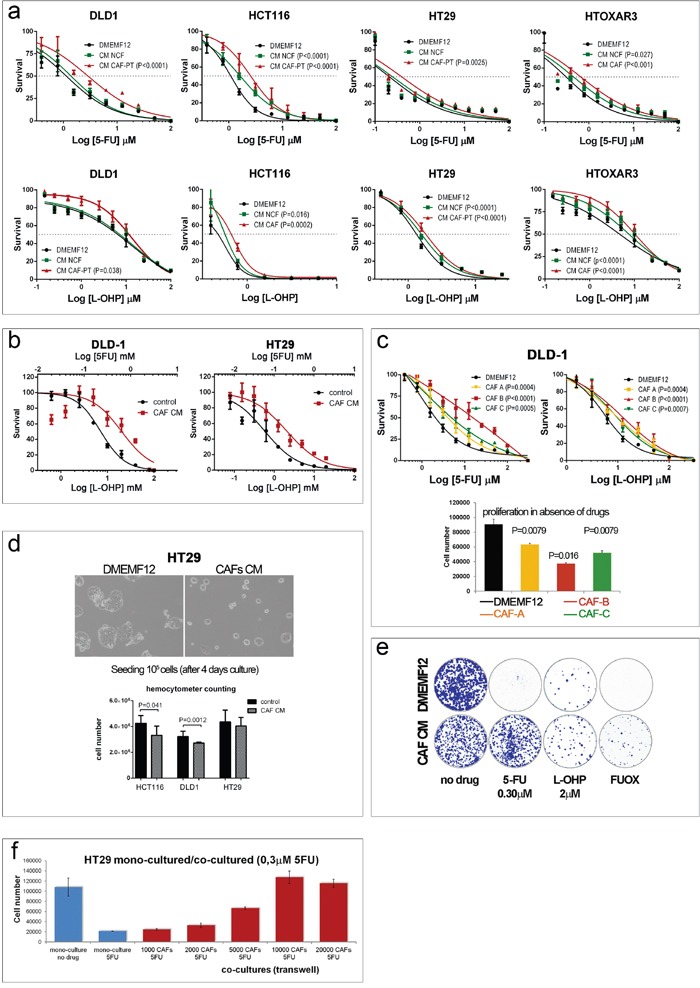
**a.** Dose-response curves of different colorectal cell lines for oxaliplatin and 5FU after 96 hours in culture in standard medium (DMEMF12), conditioned medium (CM) from normal colonic fibroblasts (NCFs) or conditioned medium from carcinoma-associated fibroblasts (CM-CAF). Values of P<0.05 were considered statistically significant (sum of squares F-test for LogIC50. **b.** Dose-response curves of DLD-1 cells (left panel) and HT29 cells (right panel) for the combination of 5FU and oxaliplatin (FUOX) cultured with DMEMF12 (control) or CAF-CM. **c.** Dose-response curves of DLD-1 cells cultured with different CAF-CM. The degree of protection conferred by CAFs is variable, probably depending on the ability to secrete particular cytokines/soluble factors that induce chemoprotection. This effect might be associated with the proliferative rate of cells in the different CMs, as depicted in the histograms in the absence of drugs (lower panel). **d.** This fact was confirmed by means ofa hemocytometer count (lower panel) after 4 days in culture, as depicted in the microphotograph. **e.** A similar diminished proliferative rate was also observed by means of a colony formation assay in the absence of drugs. However, the protective effect of CAF CM on HT29 cells was apparent after the addition of oxaliplatin and 5FU, separately or in combination. **f.** The protective effect might be involved in the concentration of the soluble factors responsible for such effect, since increasing quantities of fibroblasts increased the viability of tumor cells in a Transwell coculture proliferation assay. In addition, CAFs can induce such an effect in the presence of chemotherapy.

We checked whether CAF-soluble factors influenced the chemosensitivity of different colorectal cancer cell lines with different genetic backgrounds to the conventional anticancer drugs oxaliplatin and 5FU. We obtained the IC50 after 96 hours of continuous exposure to drugs in the presence of standard culture medium (DMEMF12) or conditioned medium (CM) from normal colonic fibroblasts (NCFs) or paired CAFs. As illustrated in Figure 1a, for all cell lines tested, CM from CAFs (CAF-CM) conferred a survival advantage on the two anticancer agents separately in relation to DMEMF12, and in combination (FUOX; Figure 1b).

In addition, as shown in Figure 1c (top panel), the degree of protection conferred by CAF-soluble factors is variable, and it will probably depend on the capacity of each CAF to secrete protective factors, suggesting a certain degree of CAF heterogeneity that we [9] and others [10] have previously reported.

We suspected that such a protective effect might be a consequence of a slower proliferative capacity of cell lines in the presence of CAF-CM, as depicted in Figure 1c (lower panel; wst-1 assay), an observation already reported by us [11] and corroborated in Figure 1d (microphotography and hemocytometer counts).

Regarding the colony-forming assay, such differential proliferative rates were also observed (bigger colonies in DMEMF12) when cells were cultured in the absence of drugs (Figure 1e, left plates), while the number of colonies (>50 cells) was similar for both conditions (DMEMF12 and CM). Conversely, CAF-CM clearly stimulated the formation of colonies in the presence of 5FU and, to a lesser extent, for oxaliplatin or the combination (FUOX).

Additionally, CAFs were capable of inducing chemoresistance even when they were also exposed to chemotherapy in a 24-well Transwell coculture system, as depicted in Figure 1f: the more fibroblasts that interact in the coculture, the more protection was observed for tumor cells, suggesting that higher concentrations of soluble factors can induce more protection.

### Cell death assessment and cell cycle phase distribution of CRC cells cultured with CAF-CM or DMEMF12 in the presence of oxaliplatin or 5FU

We first assessed the capacity of CAF-soluble factors to diminish the apoptotic rates of colorectal cancer cells lines. Surprisingly, all the cell lines tested displayed a very low percentage of cells in apoptosis when exposed to 5FU or oxaliplatin at corresponding IC50 doses (Supplementary Figure S1), as reported by other authors [12, 13]. Remarkably, the apoptotic rate was slightly lower even than under basal conditions, suggesting that, although CAF-soluble factors reduce apoptosis in tumor cells, the mechanism of cell death is something other than apoptosis. Another factor that attracted our attention was the presence of swollen cells, particularly in the presence of 5FU, that despite also being reduced with respect to the entire population occur in a lower percentage in CAF-CM than in DMEMF12 for both treatments (Supplementary Figure S1). This is probably due to some cells dying by the process of oncosis, which is characterized by nuclear and cytoplasmic swelling. Nevertheless, the total number of oncotic or apoptotic cells was low when exposed to IC50 values of both drugs, suggesting a different type of cell death or a delayed or asynchronous process. Thus, we evaluated the total population of dead cells (PI staining) for a longer exposure to 2× or 4× IC50 values for 5FU and L-OHP. As illustrated in Table 1, after 48 hours continuous exposure to 5FU or oxaliplatin, both cell types (HT29 and DLD-1) exhibited relatively low levels of PI-positive cells to a similar extent for CAF-CM and DMEMF12, even with a 4× IC50 drug concentration. However, the protective effect of CAF-CM was observed after extending the assay time until 120 hours of continuous exposure to drugs (HT29 cells) or even longer periods (168 hours for DLD-1 cells).

**Table 1:** Percentage of necrotic cells (mean of three independent experiments) measured at different times after drug administration by flow cytometry (propidium iodide staining) in cells cultured with standard DMEMF12 or CAF-conditioned media

	HT29 cells					DLD-1 cells				
	*48 hours*		*120 hours*		*48 hours*		*120 hours*		*168 hours*	
	DMEMF12	CAF-CM	DMEMF12	CAF-CM	DMEMF12	CAF-CM	DMEMF12	CAF-CM	DMEMF12	CAF-CM
**No drug**	11.16	10.77	21.5	21.5	6.24	5.5	9.61	10.3	13.5	9.3
**5FU 2x**	13.41	15.6	87	60.9^*^	6.7	4.8	7.6	7.7	13.7	9.3
**5FU 4x**	20.45	25	94.92	86^*^	6.1	6.1	8.1	11.1	21.3	19.1
**L-OHP 2x**	9.48	9.7	39.6	35.3	4.3	4.4	17.6	8.8^*^	34	17.8^*^
**L-OHP 4x**	11.4	10.5	42.1	32.2^*^	5.04	4.4	16.8	9.6^*^	64.2	40.3^*^

5FU and L-OHP were administered 24 hours after seeding at 2× or 4× IC50 of each cell type

* P<0.05, Mann-Whitney U statistic.

Given the long time required for effective cell death observed in previous experiments we aimed to evaluate the proportion of cells in each cell cycle phase to assess the effect of CAF-CM on the cell cycle perturbation induced by L-OHP or 5FU. Results are illustrated in Supplementary Table S1, confirming a less number of cells accumulated in S-phase and G_2_/M for 5FU and L-OHP respectively for HT29 cells and fewer cells in aneuploidy for DLD-1 cells, interline variation already reported for antimitotic drugs [14]. Interestingly, CAF-CM induced an accumulation of cells in G_0_G_1_, suggesting a prolonged G_1_ transit, in concordance with the lower proliferative capacity observed.

### CHK2 is overstimulated in colorectal cancer cell lines cultured with CAF-conditioned media

**Figure 2:**
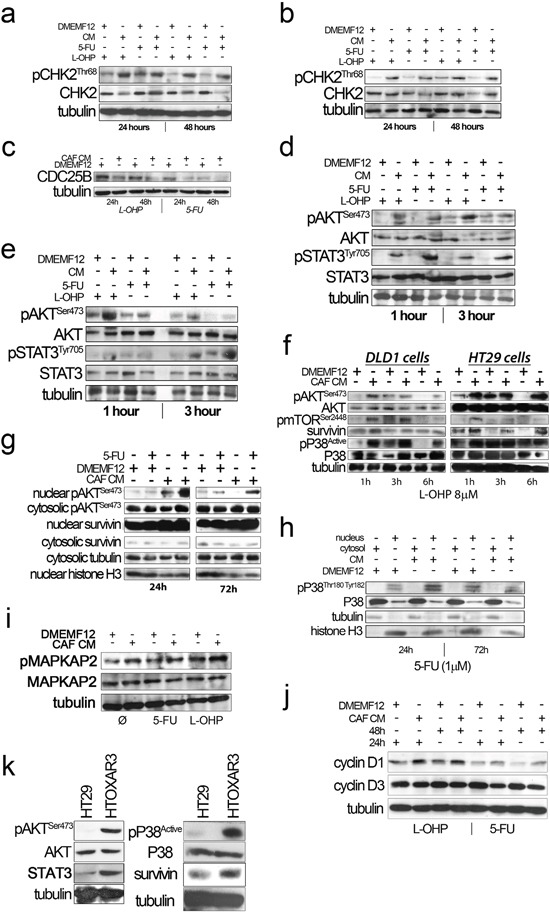
**a.** and **b.** CHK2 phosphorylation (Thr68) in the presence of CAF-CM upon oxaliplatin and 5FU administration for 24 and 48 hours in DLD-1 (A) and HCT116 (B) cell lines, respectively. **c.** Overexpression of CDC25B in DLD-1 cells treated with standard medium (DMEMF12) in the presence of drugs for 24 and 48 hours. **d** and **e.** AKT and STAT3 were phosphorylated after drug exposure (1 and 3 hours) in the presence of CAF-CM in DLD-1 and HCT116, respectively. **f.** mTOR^Ser2448^ and P38 were also differentially phosphorylated after CAF-CM treatment relative to standard medium and Survivin western blot analysis. Survivin was differentially overexpressed after culturing cells with CAF-CM. **g.** Protein extracts from nuclear and cytosolic fractions of HT29 cells treated with 5FU for 24 hours and 72 hours. We observed pAKT nuclear translocation in cells cultured in conditioned media from CAFs. Survivin was basically translocated in both standard medium and CAF-CM, although we observed overexpression under the influence of soluble factors from CAFs. **h.** Similarly, nuclear translocation of active P38 of HT29 cells was observed after 24 hours and 72 hours of treatment in the presence of CM-CAFs. **i.** A slight increase of MAPKAP2 phosphorylation, a P38 downstream kinase, was noted in HT29 cells after CAF-CM treatment. **j.** Cyclin D1 and Cyclin D3 expression in the presence of oxaliplatin and 5FU in HT29 cultured in DMEMF12 and CM-CAFs for 24 hours and 48 hours. Cyclin D1 was overexpressed in the presence of CM-CAFs in oxaliplatin and 5FU treatments. On the other hand, there was an inverse correlation between Cyclin D1 and Cyclin D3 expression levels, as previously reported (Zhang, P. et al BMC Cancer 2006, 15(6):224). **k.** HT29 and oxaliplatin-resistant derivative (HTOXAR3) cell lines cultured in the absence of drugs. In the resistant-derivative cells, pAKT, pP38, STAT3 and Survivin were overexpressed relative to sensitive cells.

The absence of apoptosis and the prolonged time needed to observe moderate levels of necrosis suggested that cells could be arrested at the point of entering mitosis with or without repairing the DNA damage induced by oxaliplatin and 5FU. CHK2 provides a survival signal for tumor cells when exposed to DNA damaging agents [15], especially for those inducing DSB, like doxorubicin, cisplatin [16], oxaliplatin and 5FU [17, 18] and is a key protein for DNA repair mediating BRCA2 phosphorilation that leads to disruption of RAD51-BRCA2 complex and then DSB repair. CHK2 has also been involved in base excision repair. In addition, CHK2 is an essential protein for maintaining genome integrity and is involved in the control of mitosis progression and repression of cell death during mitosis. Thus, we hypothesize that during mitosis CAF-soluble factors might mitigate the mitotic catastrophe induced by the drugs activating CHK2 for the proper control of the G2/M checkpoint. We observed a higher level of Thr68 phosphorylation of CHK2 in DLD-1 cells cultured in CAF-CM compared with standard DMEMF12 after long-term exposure (24 hours and 48 hours) to 5FU and oxaliplatin, as depicted in Figure 2a. We corroborated the results using a P53 wt cell line (HCT116; Figure 2b). Thus, soluble factors secreted by CAFs might induce CHK2-mediated cell cycle arrest before entering mitosis in order to ensure DNA repair, thereby preparing cells for correct cell division. Higher levels of Aurora B in CAF CM also points to a correct mitosis in such conditions, particularly for 5FU treatment, where there was a statistically significant increase of AURKB positive cells (P<0.0001) and overexpression for L-OHP (Supplementary figure S2).

The mechanisms by which cells are able to restart the cell cycle and enter mitosis after the activation of the G2/M checkpoint seem to be regulated by CDC25 phosphatases, in particular CDC25B. The latter is an essential protein that enables a cell to resume the cell cycle after DNA damage-induced cell cycle arrest [19]. When we examined the expression of CDC25B we observed overexpression of the phosphatase in cells cultured in standard culture medium compared with CAF-CM in the presence of both oxaliplatin and 5FU. This suggests a premature G2/M transition to overcome a deficient checkpoint control (Figure 2c).

### CAF-conditioned media stimulate AKT, Survivin and P38 nuclear translocation

It is well known that AKT is crucial to overcoming the G2/M cell cycle checkpoint after DNA damage. In addition, the PI3KCA/AKT/mTOR/Survivin pathway has been associated with 5FU, cisplatin and resistance to other DNA-damaging agents in many types of cancer. In evaluating the phosphorylation of AKT, we observed an increase in cells cultured with CAF-CM compared with DMEMF12 for DLD-1 (Figure 2d) and HCT116 (Figure 2e) exposed to 5FU or L-OHP. This result might be predictable, since the large quantity of ligands secreted by CAFs that activated the PI3KCA/AKT pathway as well as JAK/STAT, a cytokine-addicted pathway. Additionally, phosphorylation of Ser2448 mTOR, P38 activation and overexpression of Survivin were also evidenced in cells cultured with CAF CM (Figure 2f).

**Figure 3:**
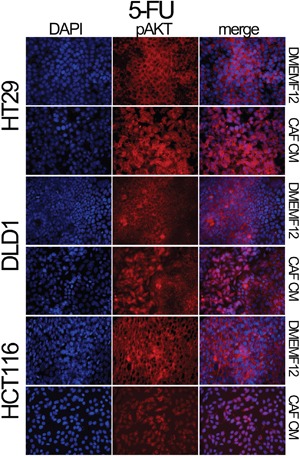
Localization of pAKT assessed by immunofluorescence HT29, DLD-1 and HCT116 cell lines were treated with 5FU in the presence of DMEMF12 or CM-CAFs. The nuclear translocation of pAKT occurs in the treatments where soluble factors from CAFs were present, while, in DMEMF12 treatments, pAKT was mainly founded predominantly in cytosolic areas.

**Figure 4:**
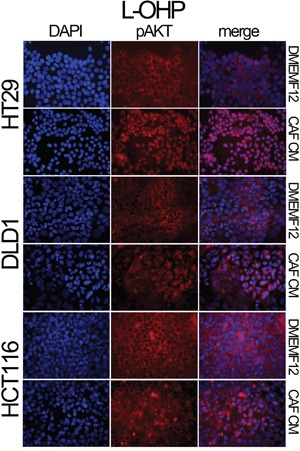
Localization of pAKT assessed by immunofluorescence HT29, DLD-1 and HCT116 cell lines were treated with oxaliplatin in the presence of DMEMF12 or CM CAFs. pAKT is translocated to the nuclei in the presence of conditioned media from CAFs. However, in DMEMF12 treatments, pAKT was predominantly located in the cytosol.

We also explored the AKT and Survivin subcellular localization. We observed sustained AKT phosphorylation in the nucleolus of cells cultured in CAF-CM (Figure 2g). This is accompanied by the nuclear translocation of Survivin and P38 (Figure 2h), which occurs more efficiently in cells under the influence of CAF-soluble factors. AKT, Survivin and P38 nuclear translocation were corroborated in different cell lines and treatments by immunofluorescence (Figures 3, 4 and Supplementary Figures S3 and S4a). Subconfluent continuous cell cultures also showed an increase in P38 downstream kinase MAPKAP2 when cells were exposed to CAF-conditioned media (Figure 2i). Additionally, the administration of a P38 inhibitor (VX-702) sensitizes colorectal cancer cells to oxaliplatin and 5FU (Supplementary Figure S4b).

We also checked the expression of Cyclin D1, as a common downstream effector of JAK/STAT and PI3KCA/AKT pathways, inducer of chemoresistance in many tumor types and essential for cell cycle progression. As illustrated in Figure 2j, cyclin D1 was overexpressed in cells cultured in CM. Additionally, there was an inverse correlation with the levels of cyclin D3 also reported by Zhang P et al [20] in relation to cisplatin sensitivity.

All the events described so far seem to involve some factors secreted by CAFs in mediating *de novo* chemoresistance to L-OHP and 5FU. All the studied pathways are also overstimulated in cells with acquired resistance, as depicted in Figure 2k, suggesting that there is a degree of convergence between stroma-mediated and genetically acquired resistance.

### STAT3 silencing sensitizes cells to oxaliplatin and 5FU. Pharmacological inhibition of AKT antagonizes with chemotherapy

**Figure 5:**
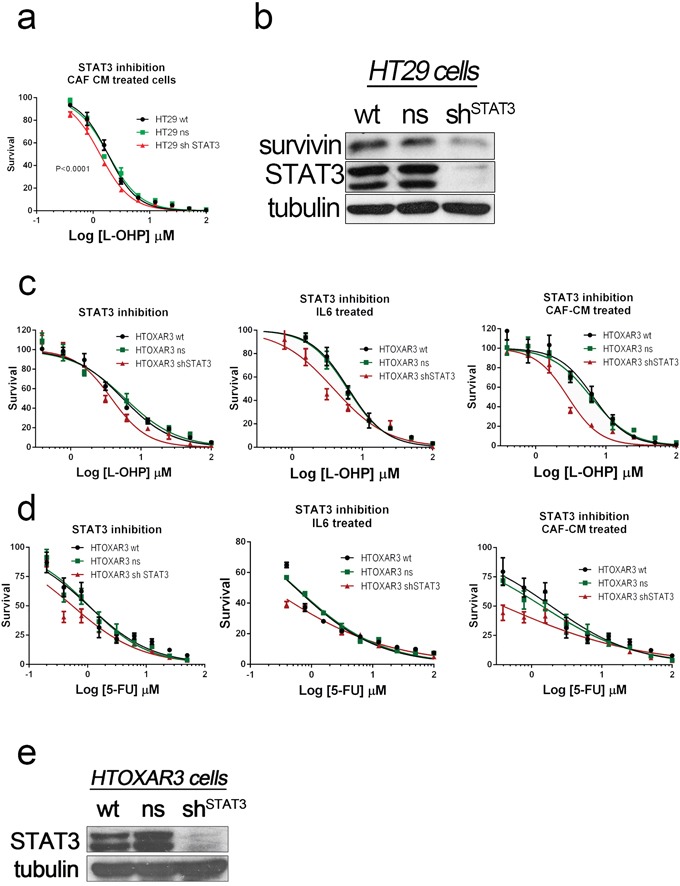
STAT3 was silenced in HT29 cells and oxaliplatin-resistant HT29 derivatives (HTOXAR3) by using shRNA STAT3-silenced HT29 cells were more sensitive to both oxaliplatin **a.** and 5FU (data not shown) than wild-type and non-silencing HT29 cells. The silencing also affected STAT3 target genes like BIRC5 according to the decrease levels of Survivin in these STAT3-silenced cells **b.** In addition the STAT3 silencing was also effective in cells with genetically determined acquired resistance to L-OHP like HTOXAR3 cells as shown in dose-response curves (panel **c.** and 5FU panel **d.** using standard conditions (DMEMF12) or particular stimulation of JAK/STAT pathway (supplemented with IL6 [10 ng/ml]) or different signaling pathways with CAF's CM. Successful silencing of total STAT3 was also demonstrated by western blot analysis in continuous subcultures of HTOXAR3 cells in CAF-CM **e.**

We have previously shown that CAF-CM can shift the IC50 curves for 5FU and oxaliplatin even in genetically determined oxaliplatin-resistant HTOXAR3 cells. Additionally, ligands present in CAF-CM were particularly efficient in activating JAK/STAT and AKT pathways, as shown in Supplementary Figure S5 and S6. Thus we decided first to check whether STAT3 inhibition could bypass the effect triggered by soluble factors released by CAFs. Stable silencing of the transcription factor by means of shRNA revealed that cells were more sensitive to oxaliplatin even in the presence of CAF's CM, where different JAK/STAT activators are present (Figure 5a). Interestingly, levels of Survivin were also decreased in STAT3 deficient cells (Figure 5b). In addition, the STAT3 silencing was also efficient sensitizing oxaliplatin-resistant cells (HTOXAR3), to both oxaliplatin and 5FU in different experimental conditions with JAK/STAT ligands (Figure 5c and 5d).

### Pharmacological inhibition of AKT antagonizes with chemotherapy

Furthermore, we also examined whether the pharmacological inhibition of AKT with MK2206, a highly selective allosteric AKT inhibitor, could also overcome the effect induced by CAF-CM.

**Figure 6:**
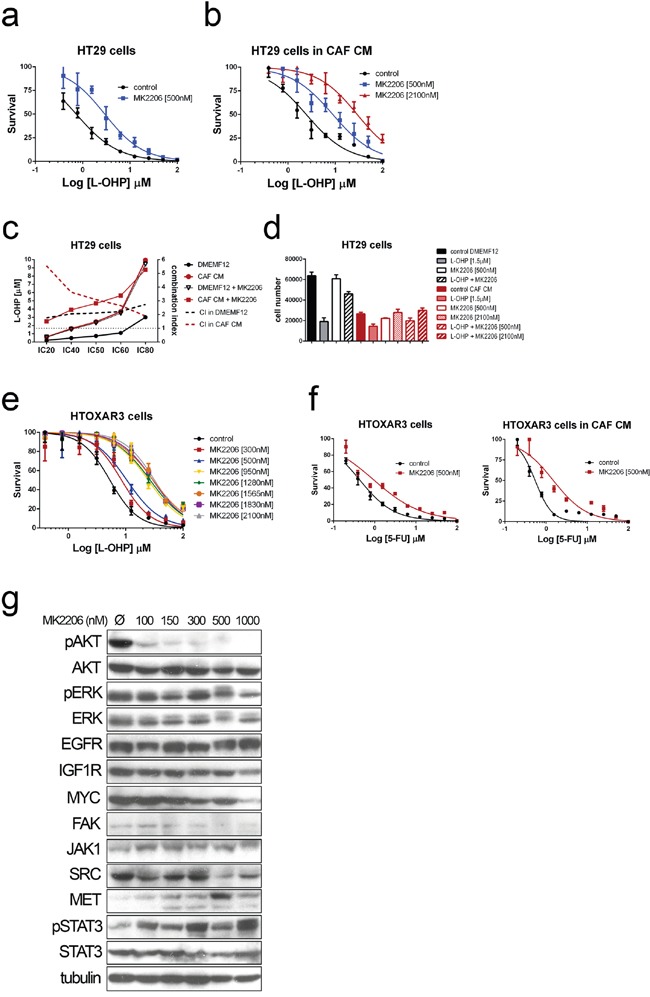
Dose-response curves for Oxaliplatin in HT29 cells cultured in DMEMF12 a. and CAF-CM b. in combination with AKT inhibitor MK2206 at IC5 and IC50 doses as single agent (500 nM or 2100 nM) **c.** The integrated dose-reduction (left Y axis) and Fa-combination index (right Y axis) plots show that combination index is >1, indicating the antagonistic effect of the combination and the increase of L-OHP concentration needed for different IC's when adding AKT inhibitor both culturing cells with standard medium (black lines) or CAF's CM (red lines), same observation also shown as histograms **d.** Dose-response curves of HTOXAR3 cells (oxaliplatin-resistant HT29 derivative) for oxaliplatin and oxaliplatin plus different concentrations of MK2206, illustrating the shift in IC50 curves associated with the antagonistic effect, the more AKT inhibitor, the more oxaliplatin to kill the 50% of cells **e.** The same results than in (a) and (b) were obtained for 5FU **f.** Immunoblots from HTOXAR3 cells cultured for 24 hours with various doses of MK2206. AKT was clearly inhibited even at very low doses (100 nM). No RTK (EGFR or IGF1R) or MYC expression changes were observed, contrary to what has been reported in other tumor types. However, we observed an inverse correlation in overexpression of JAK1 and MET in relation to the concentration of the AKT inhibitor. This seems to be accompanied by activation of STAT3 as a compensatory mechanism **g.**

Astonishingly, MK2206 seems to have an antagonistic effect with L-OHP and 5FU even at different doses tested (IC5 and IC50) for the two conditions tested (Figure 6a and 6b). The combination index values (>1) confirmed the antagonistic effect of MK2206 with oxaliplatin (Figure 6c) and for 5FU (Figure 6f). In addition there is a high correlation between the dose of MK2206 and the increase of the IC50 values for chemotherapeutic agents (Figure 6e).

We suspected that these observations could be due to compensatory mechanisms that are the consequence of inhibiting a crucial oncogenic pathway like PI3K/AKT. The expression of MYC and RTK (particularly IGF1R, EGFR and ERBB3) is involved in compensatory mechanisms after PI3K/AKT inhibition [21–24]. After 24 hours of treatment we observed potent AKT inhibition even at very low doses (100 nM; Figure 6g), confirming the efficacy of the inhibitor. Interestingly, although we did not observe overexpression of previously reported RTK or MYC, a compensatory feedback loop seems to me mediated by JAK/STAT pathway, regarding the overexpression of JAK1 and hyperactivation of STAT3 according to the dose increase of AKT inhibition (Figure 6g).

## DISCUSSION

While many research publications have described cell-autonomous processes playing decisive roles in mediating acquired resistance to chemotherapy, little is known about the influence of the tumor microenvironment on responses to chemotherapy.

The present study showed how soluble factors secreted by CAFs trigger a cell signaling cascade that protects cells from the action of conventional chemotherapy used in colorectal cancer treatment, a process involving the nuclear translocation of AKT and P38 and the activation of the JAK/STAT pathway.

It is intuitive that cytokines, chemokines and growth factors secreted in large quantities by CAFs will activate signaling cascades mainly through the PI3KCA/AKT and JAK/STAT pathways, and that such signaling is a requisite and a key event for cell proliferation, migration and invasion [25, 26]. However, these pathways are also relevant for cell cycle regulation, survival and DNA repair [27, 28, 29, 30, 31, 32].

Our results confirmed that the PI3K-AKT and JAK-STAT pathways were overexpressed in the presence of conditioned media from CAFs, even in the presence of 5FU or oxaliplatin administration. However, the location of activated AKT is crucially important for such a protective effect conferred by CAF-soluble factors. Once activated, different AKT isoforms translocate to diverse subcellular compartments to exert specific functions depending on the location [27]. Nuclear translocation of AKT during G1 in particular has been associated with cell cycle progression [33], Cyclin D1 expression [34] and DNA repair after double-strand breaks (DSBs) [35]. Additionally, different growth factors and soluble molecules have been reported to induce nuclear translocation of AKT (e.g., IGF1, insulin and NGF) [36, 37, 38]. In the context of our results, soluble factors secreted by CAFs induce nuclear AKT translocation that might provide protection from cell death, ensuring correct DNA repair and triggering cell cycle arrest to strictly control the G2/M transition caused by the increased levels of CHK2 phosphorylation in CAF-CM-cultured colorectal cancer cells. Conversely, cells grown without the contribution of cytokines and growth factors provided by the fibroblasts, in the presence of DNA-damaging agents, cannot efficiently repair the damaged DNA since the checkpoint control does not work properly. The elevated levels of CDC25B boost entry into mitosis without ensuring genome integrity. Our cell cycle analyses corroborate this. Therefore, the decision of cells to remain in the G2/M phase or to go through defective mitosis is favored by microenvironmental factors, enhancing both the nuclear translocation of AKT and the phosphorylation of CHK2. Furthermore, DNA damage-induced activation of CHK2 results in Survivin trafficking from the mitochondria to the cytosol, inducing the inhibition of apoptosis and promoting tumor cell survival [15]. In our experiments there was no suggestion that cells were dying by apoptosis. In fact, we clearly observed that cells developed a necrotic pattern after long-term exposure to both oxaliplatin and 5FU. This finding, in combination with the cytometric cell cycle analysis, led us to conclude that the higher levels of Survivin detected in whole-cell extracts of cells cultured with CAF CM might control processes other than apoptosis.

It has already been reported that Survivin can be located in different subcellular pools inside the cell, cytosol, mitochondria and nucleus [39]. These subcellular pools, as AKT, are believed to be strongly tied to its varying cellular functions. Studies have demonstrated that the nuclear pool mediates Survivin function in mitosis, while the cytosolic and mitochondrial fractions are responsible for its antiapoptotic function [40]. When we assessed the levels of Survivin in nuclear and cytoplasmic extracts we observed that almost all Survivin was translocated to the nuclei in cells cultured with CAF-CM. In addition, Survivin associates with microtubules to contribute to the correct assembly of chromosomes during the spindle formation [41]. Survivin can also repress cell death in mitotic cells, preventing mitotic catastrophe [42]. Our results suggest that nuclear AKT and STAT3 might regulate Survivin, as reported in platinated agents like cisplatin [43] and for 5-fluorouracil [44]. In fact, different cytokines and growth factors like IL6, IGF1 and CCL2 are known inducers of Survivin overexpression in a PI3-kinase/AKT or JAK/STAT dependent process [44–46]. The consequence is that many cells die during mitosis or divide inaccurately, dying in the next division or generating instability, as found in colorectal cancer cells exposed to anti-mitotic drugs [14].

Another event that is closely related to the mechanism detailed above is the translocation of P38 MAPK to the nucleus in cells cultured in CAF-CM, which occurs more efficiently than in standard culture medium. In response to DSBs induced by DNA-damaging agents, P38 is activated to control the G2/M checkpoint [47] and is translocated to the nucleus. Therefore, cytokines/chemokines or other soluble factors secreted by CAFs may enhance the nuclear translocation of P38 MAPK in tumor cells to facilitate the phosphorylation of P38 nuclear targets necessary for the induction of the appropriate G2/M cell cycle checkpoint and correct DNA repair in those cells carrying DSBs.

From the point of view of the functional assays, as a consequence of the signaling described, CRC cells cultured in CAF-CM seem to be less sensitive to chemotherapy, shifting IC50 curves to a greater extent than NCF-CM compared with standard culture medium, making it a cause of chemoresistance. All these mechanisms give rise to a slower proliferative rate, even in the absence of DNA-damaging agents, as determined by MTT assays, hemocytometer counting and prolonged G1 phase in a flow cytometric cell cycle assessment. This is in line with the fact that the subtype of colorectal cancer tumors that is most enriched in stromal components, and fibroblasts in particular, are less proliferative, overexpress Survivin, displayed fewer genetic alterations [48–50], qualities involved in chemoresistance. These are features that we observed culturing CRC cells in CAF-CM.

In addition we can not ascribe the observations and mechanisms described here to a single factor secreted by CAFs. These cells synthesize a large amount of different redundant cytokines and growth factors, most of them activating ligands of JAK/STAT or PI3KCA/AKT pathways.

Different strategies, like STAT3 silencing or AKT and P38 inhibition have been employed to diminish microenvironment-mediated drug resistance. These pathways are also involved and constitutively activated in acquired-resistance processes, suggesting convergence of stroma-induced and genetically determined resistance. The inhibition of STAT3 and P38 sensitizes cells to chemotherapy, while the inhibition of AKT elicits a compensatory feedback mechanism that appears to be mediated by the overexpression of Met and activation of STAT3 that may explain the failure of AKT inhibitors.

In conclusion, we report here that cytokines, chemokines and other soluble factors secreted by CAFs induce nuclear translocation of selected effectors that in turn slow the progress of the cell cycle, thereby affecting proliferation. In the presence of conventional chemotherapy, such effectors permit the stabilization and activation of selected downstream proteins that ensure correct DNA repair and correct mitosis entrance and exit, avoiding the damage induced by such agents. From a clinical perspective, our study provides information of use in assessing the preclinical value of STAT3 and P38 inhibition in chemotherapy-sensitizing strategies designed to overcome microenvironment-mediated resistance. Identifying the soluble factors that mediate such events might yield valuable information to circumvent microenvironmentally-mediated drug resistance, although the compensatory loops and redundancy of many such cytokines for the same signaling pathways might give rise to new resistant phenotypes. Hampering key common proteins responsible for the synthesis of such cytokines/growth factors in CAFs might be an interesting approach to avoid the protective effect exerted by such molecules and to render tumor cells sensitive to chemotherapy.

## MATERIALS AND METHODS

All the experiments were performed under the approval of the IDIBELL's Ethics Committee. Samples for fibroblast isolation were obtained under patient's informed consent.

### Fibroblast culture and conditioned media acquisition

The fibroblasts were isolated and the conditioned media (CM) were obtained as described previously [11].

### Colorectal cancer cell lines

DLD1 (MSI positive, chromosomal instability pathway negative, Kras mutated, PI3KCA mutated, TP53 mutated), HCT116 (MSI positive, chromosomal instability pathway negative, Kras mutated, PI3KCA mutated, TP53 wild type), HT29 (MSS, chromosomal instability pathway positive, Braf mutated, PI3KCA mutated, TP53 mutated), HTOXAR3 (HT29 oxaliplatin-resistant derivative; transcriptionaly characterized in GSE10405).

### Cell proliferation

WST-1 proliferation assay was performed following manufacturer instructions. Previously, 2000 cells/well (HT29, HTOXAR3, DLD-1, HCT116; all cell lines were obtained from our own cryobank except the oxaliplatin-resistant derivative HTOXAR3, kindly provided by Dr. E.M.Balibrea) were plated in six replicative wells in 96-well plates and allowed to attach, then grown in DMEM F12 at 37°C overnight. Next day, drugs were added in standard media or fibroblast CM. The cells were incubated for 5 days. The effect of the drug on each cell line in the presence or absence of CM was calculated by normalizing the number of cells after 5 days of continuous treatment to the maximum number of cells in each treatment (standard DMEMF12 medium, normal colonic fibroblast-CM [NCF-CM] or CAF-CM).

### Cell cycle analysis

The cell cycle was assessed by conventional PI staining.

The samples analyses were performed in a Gallios Flow Cytometer and the generated data analyzed using FCS Express Analysis Software (*de Novo* software).

### Colony formation assay

1×10^3^ CRC cells were plated in 60-mm culture dishes (or 2×10^2^ in 6-well plates) and incubated at 37°C. After overnight incubation, treatments were added and incubated for 2 weeks. The clones were fixed with methanol for 2 minutes, stained with 0.5% crystal violet and incubated at room temperature for 20 minutes. The cells were washed to remove staining exceeded. Colonies were defined from counts of at least 50 cells.

### Immunofluorescence staining

The cells were seeded in coverslip slides on 6-well plates, fixed with paraformaldehyde (PFA) 4% for 20 minutes and washed 3 times with PBS 1×, permeabilized with PBS 1× 0.1% Triton X-100 at room temperature for 5 minutes and blocked with BSA 2% for 45 minutes–2 hours. Cells were incubated with primary antibody in BSA 2% in a dilution 1:50 overnight and then incubated with 1:200 fluorescence secondary antibody in BSA 2% for 1 hour at room temperature. Coverslip slides were mounted in VectaShield with DAPI (Vector Laboratories, Burlingame, USA).

### Western blot analysis

Protein concentration was determined by the BCA Protein Assay (Pierce, Rockford, IL, USA). 30 μg of the protein extract was subjected to sodium dodecyl sulfate polyacrylamide gel electrophoresis (SDS-PAGE), and transferred to PVDF membranes. After blocking for 1 hour with 5% dried non-fat milk in TBS 1× - Tween 0.1%, the membranes were incubated with primary antibody diluted 1:1000 in 1% bovine serum albumin (BSA) in TBS 1× - Tween 0.1%. Antibody binding was detected using a secondary antibody diluted 1:2000 in TBS 1× - Tween 0.1% and an enhanced chemiluminescence (ECL) detection kit (Amersham, Buckinghamshire, UK). α-tubulin expression was used as an endogenous control. A general protocol for nuclear and cytosolic fractions separation was used. Briefly, cells were washed twice in PBS and scrapped in 300 μl of cytoplasmic lysis buffer containing 10 mM HEPES pH 7.4, 10 nM KCl, 0.01 mM EDTA, 0.1 mM EGTA, 2 mM dithiothreitol, 5 mM Na_2_VO_4_, 20 mM sodium beta-glycerophosphate, 0.1% Nonidet P-40, and a cocktail of protease inhibitors (Roche). Nuclei were sedimented by centrifugation and the supernatant containing the cytoplasmic fraction removed. The nuclei were then washed in 1 ml of cytoplasmic lysis buffer to remove any contaminating cytoplasm and resedimented. The nuclei were then lysed in 100l of denaturing lysis buffer.

### Cell death assessment

In order to assess necrosis or apoptosis by flow cytometry and immunofluorescence, cells were stained with the Promokine apoptotic/necrotic/healthy cell detection kit (cat #PK-CA707-30018, PromoCell, Heidelberg, Germany). First, the cells were detached from the cell culture plate using StemPro Accutase Cell Dissociation Reagent (cat #A1110501, Thermo Scientific), washed twice with 1X binding buffer and then incubated for 15 minutes at room temperature with staining solution (5 μL of FITC-Annexin V, 5 μL of Ethidium Homodimer III and 5 μL of Hoechst 33342 to 100 μL of 1× binding buffer). Cells were washed 1-2 times and mounted on a slide for microscope viewing, or resuspended in 400 μL in 1× binding buffer and then sent for flow cytometry analysis within 1 hour of staining in a Gallios Flow Cytometer (Beckman Coulter, Krefeld, Germany).

The Ac-DEVD-AMC Caspase-3 fluorogenic assay (BD Biosciences Pharmingen, San Agustin de Guadalix, Madrid, Spain) was performed to assess caspase-dependent apoptosis. Whole cellular extract (adherent cells and floating cells) was recovered with lysis buffer and, subsequently, 20 μM of Ac-DEVD-AMC and the cell lysate were replaced by 1 mL of protease assay buffer (described in the manufacturer's instructions). The reaction mixtures were incubated for 2 hours at 37°C in the dark and then measured at wavelengths of 380 nm excitation and 430-460 nm emission.

Immunofluorescence of cleaved caspase 3 was also carried out.

### Chemicals

MK-2206 2HCl (AKT inhibitor) and VX-702 (P38 inhibitor) were purchased from Selleck Chemicals (Houston, TX, USA) and dissolved in DMSO as a stock solution. CRC cells were seeded at a density of 2000/4000 cells per well in 96-well plates and incubated with DMEMF12 overnight. Inhibitors were then added to the cells in combination with 5FU or oxaliplatin.

### Silencing STAT3 by lentiviral shRNA

GIPZ lentiviral shRNA (cat #V3LHS_641817 and #V3LHS_641819 clones purchased from Thermo Scientific) was used to establish STAT3 knockdown in HTOXAR3 colorectal cell lines. Glycerol-stored plasmid was previously replicated, isolated, transfected in HEK293T cells and finally transduced in target cells. Cells were selected, according to kill curve values, with puromycin (2 μg/ml). A non-silencing GIPZ lentiviral shRNA containing a random vector was used as a control in both cell lines in subsequent experiments. Western blot was performed to confirm STAT3 silencing efficiency in colorectal cell lines.

### Statistical analysis

Data from independent experiments were statistically analyzed using the nonparametric Mann-Whitney U test to compare group differences between experimental and control samples at a level of significance of P<0.05. Extra sum-of-squares F test was carried out in GraphPad PRISM 6 (GraphPad Software, San Diego, California, USA) to identify statistically significant differences (P<0.05) between IC50 curves.

## SUPPLEMENTARY DATA FIGURES AND TABLE





## References

[1] Cammarota R, Bertolini V, Pennesi G, Bucci EO, Gottardi O, Garlanda C, Laghi L, Barberis MC, Sessa F, Noonan DM, Albini A (2010). The tumor microenvironment of colorectal cancer: stromal TLR-4 expression as a potential prognostic marker. J Transl Med.

[2] Cheah PY (2009). Recent advances in colorectal cancer genetics and diagnostics. Crit Rev Oncol Hematol.

[3] Wernyj RP, Morin PJ (2004). Molecular mechanisms of platinum resistance: still searching for the Achilles' heel. Drug Resist Updat.

[4] Meads MB, Gatenby RA, Dalton WS (2009). Environment-mediated drug resistance: a major contributor to minimal residual disease. Nat Rev Cancer.

[5] Nagasaki T, Hara M, Nakanishi H, Takahashi H, Sato M, Takeyama H (2014). Interleukin-6 released by colon cancer-associated fibroblasts is critical for tumour angiogenesis: anti-interleukin-6 receptor antibody suppressed angiogenesis and inhibited tumour-stroma interaction. Br J Cancer.

[6] Hazlehurst LA, Landowski TH, Dalton WS (2003). Role of the tumor microenvironment in mediating de novo resistance to drugs and physiological mediators of cell death. Oncogene.

[7] Castells M, Thibault B, Delord JP, Couderc B (2012). Implication of tumor microenvironment in chemoresistance: tumor-associated stromal cells protect tumor cells from cell death. Int J Mol Sci.

[8] Straussman R, Morikawa T, Shee K, Barzily-Rokni M, Qian ZR, Du J, Davis A, Mongare MM, Gould J, Frederick DT, Cooper ZA, Chapman PB, Solit DB (2012). Tumour micro-environment elicits innate resistance to RAF inhibitors through HGF secretion. Nature.

[9] Berdiel-Acer M, Sanz-Pamplona R, Calon A, Cuadras D, Berenguer A, Sanjuan X, Paules MJ, Salazar R, Moreno V, Batlle E, Villanueva A, Mollevi DG (2014). Differences between CAFs and their paired NCF from adjacent colonic mucosa reveal functional heterogeneity of CAFs, providing prognostic information. Mol Oncol.

[10] Herrera M, Islam AB, Herrera A, Martin P, Garcia V, Silva J, Garcia JM, Salas C, Casal I, de Herreros AG, Bonilla F, Pena C (2013). Functional heterogeneity of cancer-associated fibroblasts from human colon tumors shows specific prognostic gene expression signature. Clin Cancer Res.

[11] Berdiel-Acer M, Bohem ME, Lopez-Doriga A, Vidal A, Salazar R, Martinez-Iniesta M, Santos C, Sanjuan X, Villanueva A, Mollevi DG (2011). Hepatic carcinoma-associated fibroblasts promote an adaptative response in colorectal cancer cells that inhibit proliferation and apoptosis: nonresistant cells die by nonapoptotic cell death. Neoplasia.

[12] William-Faltaos S, Rouillard D, Lechat P, Bastian G (2006). Cell cycle arrest and apoptosis induced by oxaliplatin (L-OHP) on four human cancer cell lines. Anticancer Res.

[13] Gines A, Bystrup S, Ruiz de Porras V, Guardia C, Musulen E, Martinez-Cardus A, Manzano JL, Layos L, Abad A, Martinez-Balibrea E (2015). PKM2 Subcellular Localization Is Involved in Oxaliplatin Resistance Acquisition in HT29 Human Colorectal Cancer Cell Lines. PLoS One.

[14] Gascoigne KE, Taylor SS (2008). Cancer cells display profound intra- and interline variation following prolonged exposure to antimitotic drugs. Cancer Cell.

[15] Ghosh JC, Dohi T, Raskett CM, Kowalik TF, Altieri DC (2006). Activated checkpoint kinase 2 provides a survival signal for tumor cells. Cancer Res.

[16] Vakifahmetoglu H, Olsson M, Zhivotovsky B (2008). Death through a tragedy: mitotic catastrophe. Cell Death Differ.

[17] Adamsen BL, Kravik KL, De Angelis PM (2011). DNA damage signaling in response to 5-fluorouracil in three colorectal cancer cell lines with different mismatch repair and TP53 status. Int J Oncol.

[18] Nakagawa Y, Kajihara A, Takahashi A, Kondo N, Mori E, Kirita T, Ohnishi T (2014). The BRCA2 gene is a potential molecular target during 5-fluorouracil therapy in human oral cancer cells. Oncol Rep.

[19] van Vugt MA, Bras A, Medema RH (2004). Polo-like kinase-1 controls recovery from a G2 DNA damage-induced arrest in mammalian cells. Mol Cell.

[20] Zhang P, Zhang Z, Zhou X, Qiu W, Chen F, Chen W (2006). Identification of genes associated with cisplatin resistance in human oral squamous cell carcinoma cell line. BMC Cancer.

[21] Tan J, Li Z, Lee PL, Guan P, Aau MY, Lee ST, Feng M, Lim CZ, Lee EY, Wee ZN, Lim YC, Karuturi RK, Yu Q (2013). PDK1 signaling toward PLK1-MYC activation confers oncogenic transformation, tumor-initiating cell activation, and resistance to mTOR-targeted therapy. Cancer Discov.

[22] Chandarlapaty S, Sawai A, Scaltriti M, Rodrik-Outmezguine V, Grbovic-Huezo O, Serra V, Majumder PK, Baselga J, Rosen N (2011). AKT inhibition relieves feedback suppression of receptor tyrosine kinase expression and activity. Cancer Cell.

[23] Chakrabarty A, Sanchez V, Kuba MG, Rinehart C, Arteaga CL (2012). Feedback upregulation of HER3 (ErbB3) expression and activity attenuates antitumor effect of PI3K inhibitors. Proc Natl Acad Sci U S A.

[24] Fox EM, Kuba MG, Miller TW, Davies BR, Arteaga CL (2013). Autocrine IGF-I/insulin receptor axis compensates for inhibition of AKT in ER-positive breast cancer cells with resistance to estrogen deprivation. Breast Cancer Res.

[25] Bromberg J, Wang TC (2009). Inflammation and cancer: IL-6 and STAT3 complete the link. Cancer Cell.

[26] Fu S, Dong L, Sun W, Xu Y, Gao L, Miao Y (2013). Stromal-epithelial crosstalk provides a suitable microenvironment for the progression of ovarian cancer cells *in vitro*. Cancer Invest.

[27] Martelli AM, Tabellini G, Bressanin D, Ognibene A, Goto K, Cocco L, Evangelisti C (2012). The emerging multiple roles of nuclear Akt. Biochim Biophys Acta.

[28] Lin L, Liu A, Peng Z, Lin HJ, Li PK, Li C, Lin J (2015). STAT3 is necessary for proliferation and survival in colon cancer-initiating cells. Cancer Res.

[29] Corvinus FM, Orth C, Moriggl R, Tsareva SA, Wagner S, Pfitzner EB, Baus D, Kaufmann R, Huber LA, Zatloukal K, Beug H, Ohlschlager P, Schutz A, Halbhuber KJ, Friedrich K (2005). Persistent STAT3 activation in colon cancer is associated with enhanced cell proliferation and tumor growth. Neoplasia.

[30] Kusaba T, Nakayama T, Yamazumi K, Yakata Y, Yoshizaki A, Nagayasu T, Sekine I (2005). Expression of p-STAT3 in human colorectal adenocarcinoma and adenoma; correlation with clinicopathological factors. J Clin Pathol.

[31] Lin Q, Lai R, Chirieac LR, Li C, Thomazy VA, Grammatikakis I, Rassidakis GZ, Zhang W, Fujio Y, Kunisada K, Hamilton SR, Amin HM (2005). Constitutive activation of JAK3/STAT3 in colon carcinoma tumors and cell lines: inhibition of JAK3/STAT3 signaling induces apoptosis and cell cycle arrest of colon carcinoma cells. Am J Pathol.

[32] Tsareva SA, Moriggl R, Corvinus FM, Wiederanders B, Schutz A, Kovacic B, Friedrich K (2007). Signal transducer and activator of transcription 3 activation promotes invasive growth of colon carcinomas through matrix metalloproteinase induction. Neoplasia.

[33] van Opstal A, Bijvelt J, van Donselaar E, Humbel BM, Boonstra J (2012). Inhibition of protein kinase B activity induces cell cycle arrest and apoptosis during early G(1) phase in CHO cells. Cell Biol Int.

[34] Badve S, Collins NR, Bhat-Nakshatri P, Turbin D, Leung S, Thorat M, Dunn SE, Geistlinger TR, Carroll JS, Brown M, Bose S, Teitell MA, Nakshatri H (2010). Subcellular localization of activated AKT in estrogen receptor- and progesterone receptor-expressing breast cancers: potential clinical implications. Am J Pathol.

[35] Bozulic L, Surucu B, Hynx D, Hemmings BA (2008). PKBalpha/Akt1 acts downstream of DNA-PK in the DNA double-strand break response and promotes survival. Mol Cell.

[36] Meier R, Alessi DR, Cron P, Andjelkovic M, Hemmings BA (1997). Mitogenic activation, phosphorylation, and nuclear translocation of protein kinase Bbeta. J Biol Chem.

[37] Leinninger GM, Backus C, Uhler MD, Lentz SI, Feldman EL (2004). Phosphatidylinositol 3-kinase and Akt effectors mediate insulin-like growth factor-I neuroprotection in dorsal root ganglia neurons. FASEB J.

[38] Borgatti P, Martelli AM, Tabellini G, Bellacosa A, Capitani S, Neri LM (2003). Threonine 308 phosphorylated form of Akt translocates to the nucleus of PC12 cells under nerve growth factor stimulation and associates with the nuclear matrix protein nucleolin. J Cell Physiol.

[39] Fortugno P, Wall NR, Giodini A, O'Connor DS, Plescia J, Padgett KM, Tognin S, Marchisio PC, Altieri DC (2002). Survivin exists in immunochemically distinct subcellular pools and is involved in spindle microtubule function. J Cell Sci.

[40] Colnaghi R, Connell CM, Barrett RM, Wheatley SP (2006). Separating the anti-apoptotic and mitotic roles of survivin. J Biol Chem.

[41] Altieri DC (2006). The case for survivin as a regulator of microtubule dynamics and cell-death decisions. Curr Opin Cell Biol.

[42] O'Connor DS, Grossman D, Plescia J, Li F, Zhang H, Villa A, Tognin S, Marchisio PC, Altieri DC (2000). Regulation of apoptosis at cell division by p34cdc2 phosphorylation of survivin. Proc Natl Acad Sci U S A.

[43] Sun XP, Dong X, Lin L, Jiang X, Wei Z, Zhai B, Sun B, Zhang Q, Wang X, Jiang H, Krissansen GW, Qiao H, Sun X (2014). Up-regulation of survivin by AKT and hypoxia-inducible factor 1alpha contributes to cisplatin resistance in gastric cancer. FEBS J.

[44] Juan HC, Tsai HT, Chang PH, Huang CY, Hu CP, Wong FH (2013). Insulin-like growth factor 1 mediates 5-fluorouracil chemoresistance in esophageal carcinoma cells through increasing survivin stability. Apoptosis.

[45] Ara T, Nakata R, Sheard MA, Shimada H, Buettner R, Groshen SG, Ji L, Yu H, Jove R, Seeger RC, DeClerck YA (2013). Critical role of STAT3 in IL-6-mediated drug resistance in human neuroblastoma. Cancer Res.

[46] Roca H, Varsos Z, Pienta KJ (2008). CCL2 protects prostate cancer PC3 cells from autophagic death via phosphatidylinositol 3-kinase/AKT-dependent survivin up-regulation. J Biol Chem.

[47] Kurosu T, Takahashi Y, Fukuda T, Koyama T, Miki T, Miura O (2005). p38 MAP kinase plays a role in G2 checkpoint activation and inhibits apoptosis of human B cell lymphoma cells treated with etoposide. Apoptosis.

[48] Santos C, Sanz-Pamplona R, Nadal E, Grasselli J, Pernas S, Dienstmann R, Moreno V, Tabernero J, Salazar R (2015). Intrinsic cancer subtypes--next steps into personalized medicine. Cell Oncol (Dordr).

[49] Calon A, Lonardo E, Berenguer-Llergo A, Espinet E, Hernando-Momblona X, Iglesias M, Sevillano M, Palomo-Ponce S, Tauriello DV, Byrom D, Cortina C, Morral C, Barcelo C (2015). Stromal gene expression defines poor-prognosis subtypes in colorectal cancer. Nat Genet.

[50] Marisa L, de Reynies A, Duval A, Selves J, Gaub MP, Vescovo L, Etienne-Grimaldi MC, Schiappa R, Guenot D, Ayadi M, Kirzin S, Chazal M, Flejou JF (2013). Gene expression classification of colon cancer into molecular subtypes: characterization, validation, and prognostic value. PLoS Med.

