# Supplementary material for: Carcinoma-associated fibroblasts affect sensitivity to oxaliplatin and 5FU in colorectal cancer cells
Source: Oncotarget. 2016 Aug 8;7(37):59766–80. doi: 10.18632/oncotarget.11121 (PMC5312347; doi:10.18632/oncotarget.11121)
Supplement: Supplementary file 1 [file oncotarget-07-59766-s001.pdf]

Carcinoma-associated fibroblasts affect sensitivity to oxaliplatin and 5FU in colorectal cancer cells

SUPPLEMENTARY DATA

Colony formation assay

1×10^3 CRC cells were plated in 60-mm culture dishes (or 2×10^2 in 6-well plates) and incubated at 37°C. After overnight incubation, treatments were added and incubated for 2 weeks. The clones were fixed with methanol for 2 minutes, stained with 0.5% crystal violet and incubated at room temperature for 20 minutes. The cells were washed to remove staining exceeded. Colonies were defined from counts of at least 50 cells.

Immunofluorescence staining

The cells were seeded in coverslip slides on 6-well plates, fixed with paraformaldehyde (PFA) 4% for 20 minutes and washed 3 times with PBS 1×, permeabilized with PBS 1× 0.1% Triton X-100 at room temperature for 5 minutes and blocked with BSA 2% for 45 minutes–2 hours. Cells were incubated with primary antibody in BSA 2% in a dilution 1:50 overnight and then incubated with 1:200 fluorescence secondary antibody in BSA 2% for 1 hour at room temperature. Coverslip slides were mounted in VectaShield with DAPI (Vector Laboratories, Burlingame, USA).

Western blot analysis

Cells were plated onto different dishes at a density of 70% cells per dish and then treated according to the experimental design. After washing the cultures with PBS, cells were lysed with RIPA buffer.

Protein concentration was determined by the BCA Protein Assay (Pierce, Rockford, IL, USA). 30 µg of the protein extract was subjected to sodium dodecyl sulfate polyacrylamide gel electrophoresis (SDS-PAGE), and transferred to PVDF membranes. After blocking for 1 hour with 5% dried non-fat milk in TBS 1× - Tween 0.1%, the membranes were incubated with primary antibody diluted 1:1000 in 1% bovine serum albumin (BSA) in TBS 1× - Tween 0.1%. Antibody binding was detected using a secondary antibody diluted 1:2000 in TBS 1× - Tween 0.1% and an enhanced chemiluminescence (ECL) detection kit (Amersham, Buckinghamshire, UK). α-tubulin expression was used as an endogenous control. A general protocol for nuclear and cytosolic fractions separation was used.

Cell death assessment

In order to assess necrosis or apoptosis by flow cytometry and immunofluorescence, cells were stained with the Promokine apoptotic/necrotic/healthy cell detection kit (cat #PK-CA707-30018, PromoCell, Heidelberg, Germany). First, the cells were detached from the cell culture plate using StemPro Accutase Cell Dissociation Reagent (cat #A1110501, Thermo Scientific), washed twice with 1X binding buffer and then incubated for 15 minutes at room temperature with staining solution (5 µL of FITC-Annexin V, 5 µL of Ethidium Homodimer III and 5 µL of Hoechst 33342 to 100 µL of 1× binding buffer). Cells were washed 1-2 times and mounted on a slide for microscope viewing, or resuspended in 400 µL in 1× binding buffer and then sent for flow cytometry analysis within 1 hour of staining in a Gallios Flow Cytometer (Beckman Coulter, Krefeld, Germany).

The Ac-DEVD-AMC Caspase-3 fluorogenic assay (BD Biosciences Pharmingen, San Agustin de Guadalix, Madrid, Spain) was performed to assess caspase-dependent apoptosis. Whole cellular extract (adherent cells and floating cells) was recovered with lysis buffer and, subsequently, 20 µM of Ac-DEVD-AMC and the cell lysate were replaced by 1 mL of protease assay buffer (described in the manufacturer's instructions). The reaction mixtures were incubated for 2 hours at 37°C in the dark and then measured at wavelengths of 380 nm excitation and 430-460 nm emission.

Immunofluorescence of cleaved caspase 3 was also carried out.

AKT inhibition by oral allosteric MK2206 inhibitor and P38 by VX-702 selective P38α inhibitor

MK-2206 2HCl and VX-702 were purchased from Selleck Chemicals (Houston, TX, USA) and dissolved in DMSO as a stock solution. CRC cells were seeded at a density of 2000/4000 cells per well in 96-well plates and incubated with DMEMF12 overnight. Inhibitors were then added to the cells in combination with 5FU or oxaliplatin. Cell proliferation was determined after 5 days by the WST-1 proliferation assay. The effect of the drug on each cell line in the presence or absence of inhibitors was calculated by normalizing the cell frequency after 5 days of continuous treatment with respect to the maximum frequency of cells in each treatment. Western blot was performed to confirm inhibition of $pAKT^{Ser473}$ in colorectal cell lines.

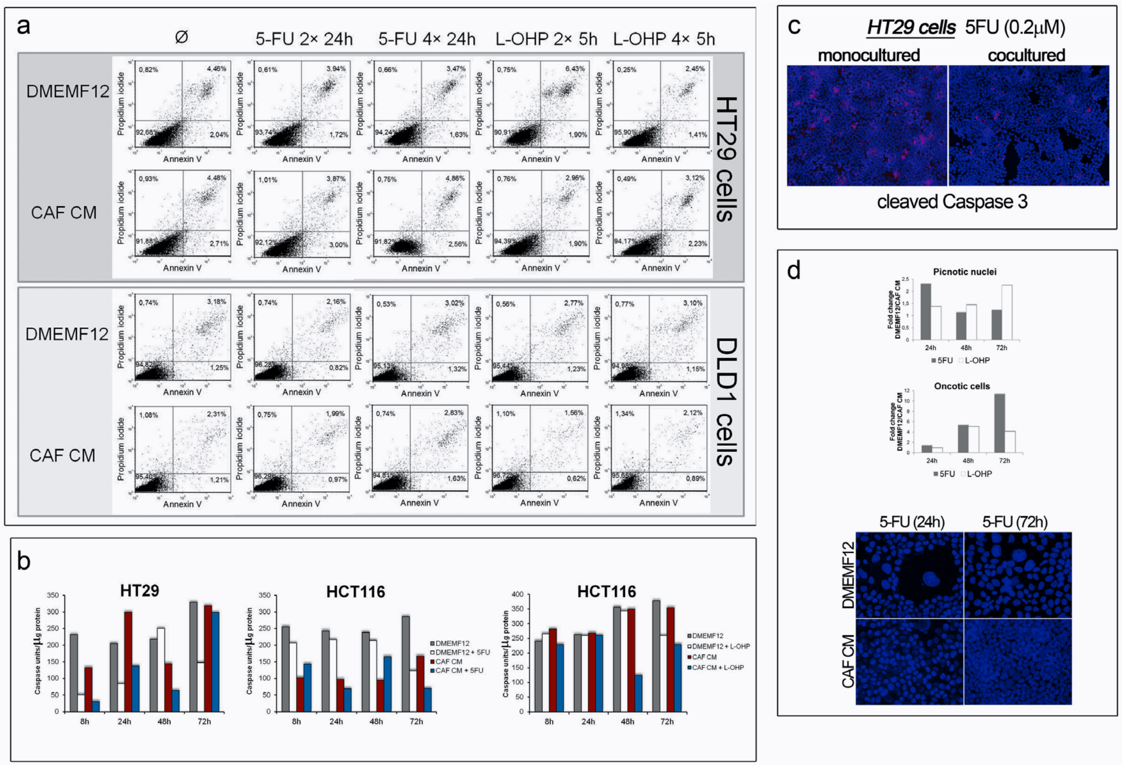

Supplementary Figure S1: a. PI and Annexin V staining by flow cytometry. HT29 and DLD-1 cell lines were treated with 2X and 4X their respective IC50 for oxaliplatin (5 hours) and 5FU (24 hours). **b.** Ac-DEVD-AMC Capase-3 fluorogenic assay. HT29 and HCT116 cell lines were treated with drugs and their caspase-3 activity was measured at 8, 24, 48 and 72 hours in the presence of standard medium or CM-CAFs. Left and middle panel cells treated with 5FU; right panel cells treated with oxaliplatin. **c.** Cleaved-caspase 3 immunofluorescence. The HT29 cell line was treated in monoculture and coculture in the presence of 0.2 μM of 5FU. A decrease in cleaved Caspase-3 staining was noted in cells cocultured with CAFs. **d.** Picnotic nuclei and oncotic cells. Bars represent the magnitude of change between cells treated with oxaliplatin or 5FU when cultured with standard medium compared with CAF-conditioned medium at different times. DAPI staining shows the oncotic morphology of cells treated with 5FU for 24 and 72 hours in DMEMF12 and CAF-CM.

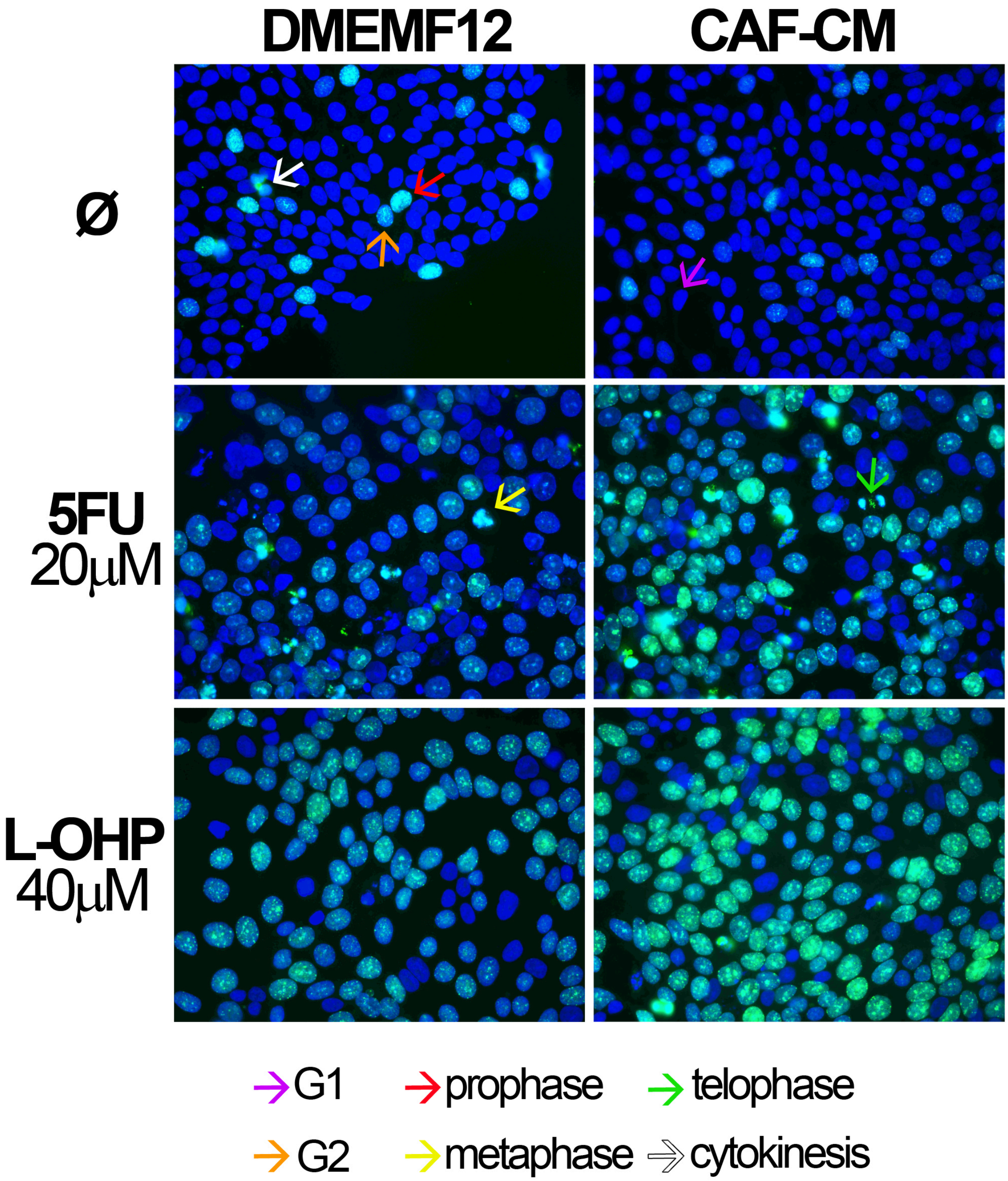

Supplementary Figure S2: Aurora B staining in cells cultured in DMEMF12 or CAF-CM in absence or presence of oxaliplatin or 5FU. The higher number of positive cells for 5FU in CAF-CM was very evident, even considering the increase of cells in late cytokinesis, suggesting a correct exit from mitosis in these cells. For oxaliplatin the number of positive cells was very similar among treatments but the intensity was clearly higher when culturing cells in CAF-CM, suggesting an overexpression of Aurora B.

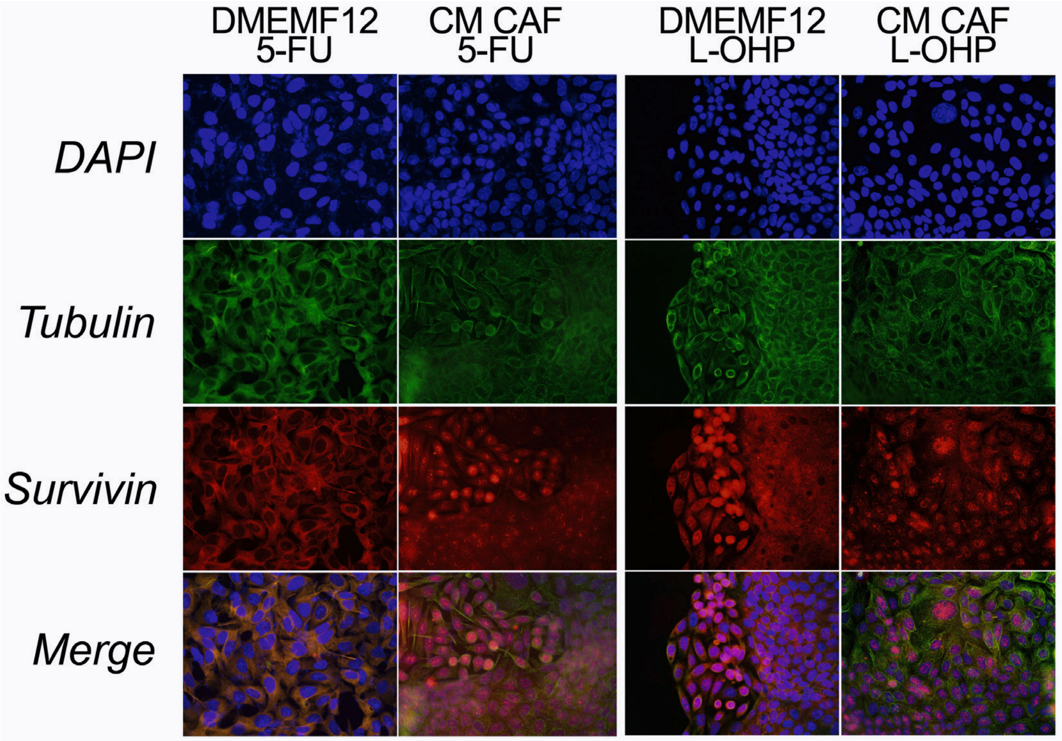

Supplementary Figure S3: Immunofluorescence of nuclear translocation of Survivin. HT29 (left panels) and HCT116 (right panels) cell lines were treated with oxaliplatin or 5FU in the presence of DMEMF12 or CM-CAFs. Survivin was mainly translocated to the nuclei in the treatments where CM-CAFs were present, while, in DMEMF12 Survivin was mainly located in cytosolic regions, but also present in the nuclei.

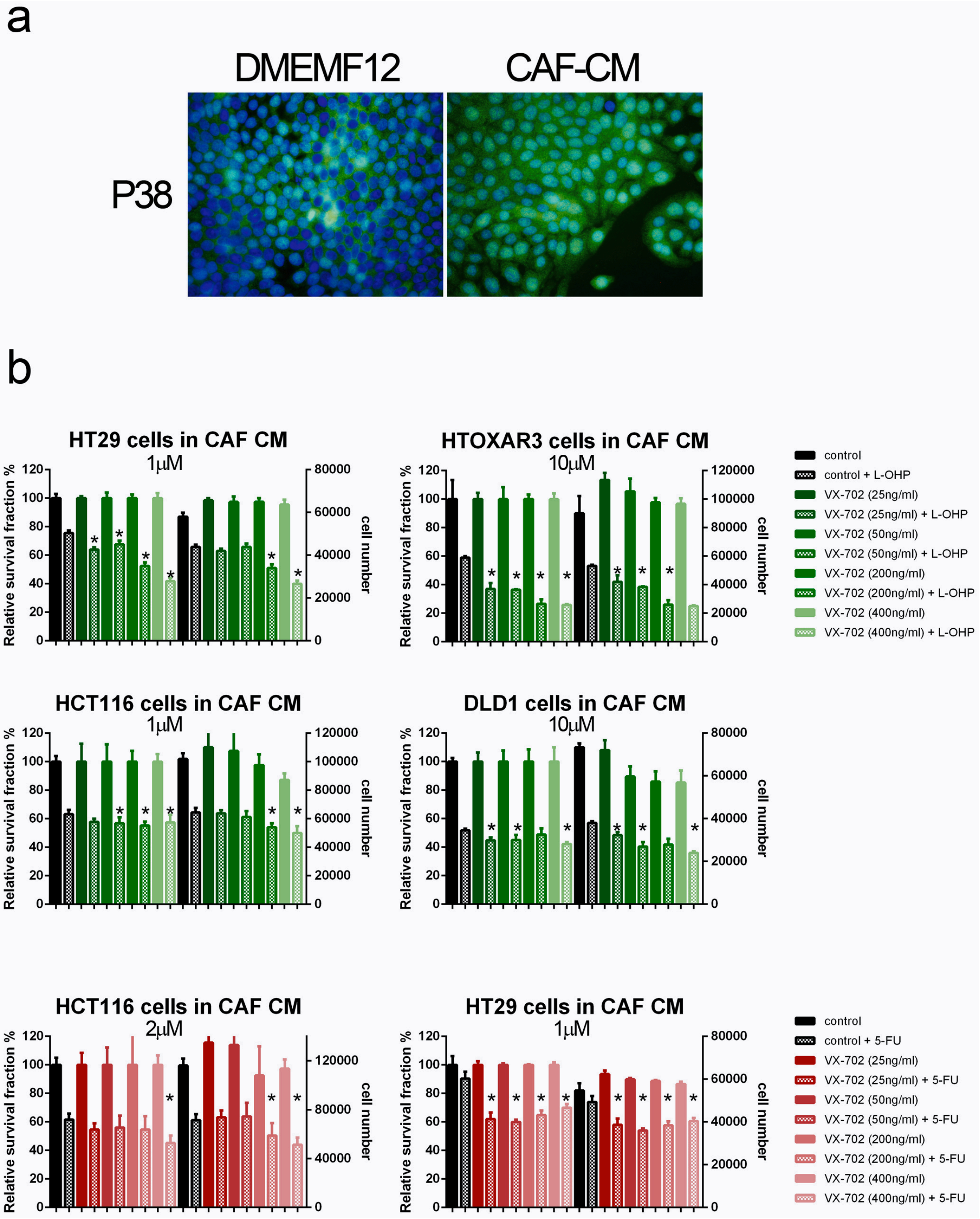

Supplementary Figure S4: a. P38 localization by immunofluorescence in DLD-1 cells. P38 was translocated to the nuclei in the presence of conditioned media from CAFs. **b.** VX-702, a p38 inhibitor, sensitizes HT29, HCT116 and DLD-1 colorectal cell lines to drugs in the presence of conditioned media from CAFs. Different concentrations of inhibitor were used in a range of 25-400 ng/ml. Relative values of the survival fraction are calculated from each VX-702 concentration relative to the corresponding VX-702 concentration without oxaliplatin or 5FU. Asterisks denote statistically significant differences between VX-702-treated groups and oxaliplatin or 5FU as single agents.

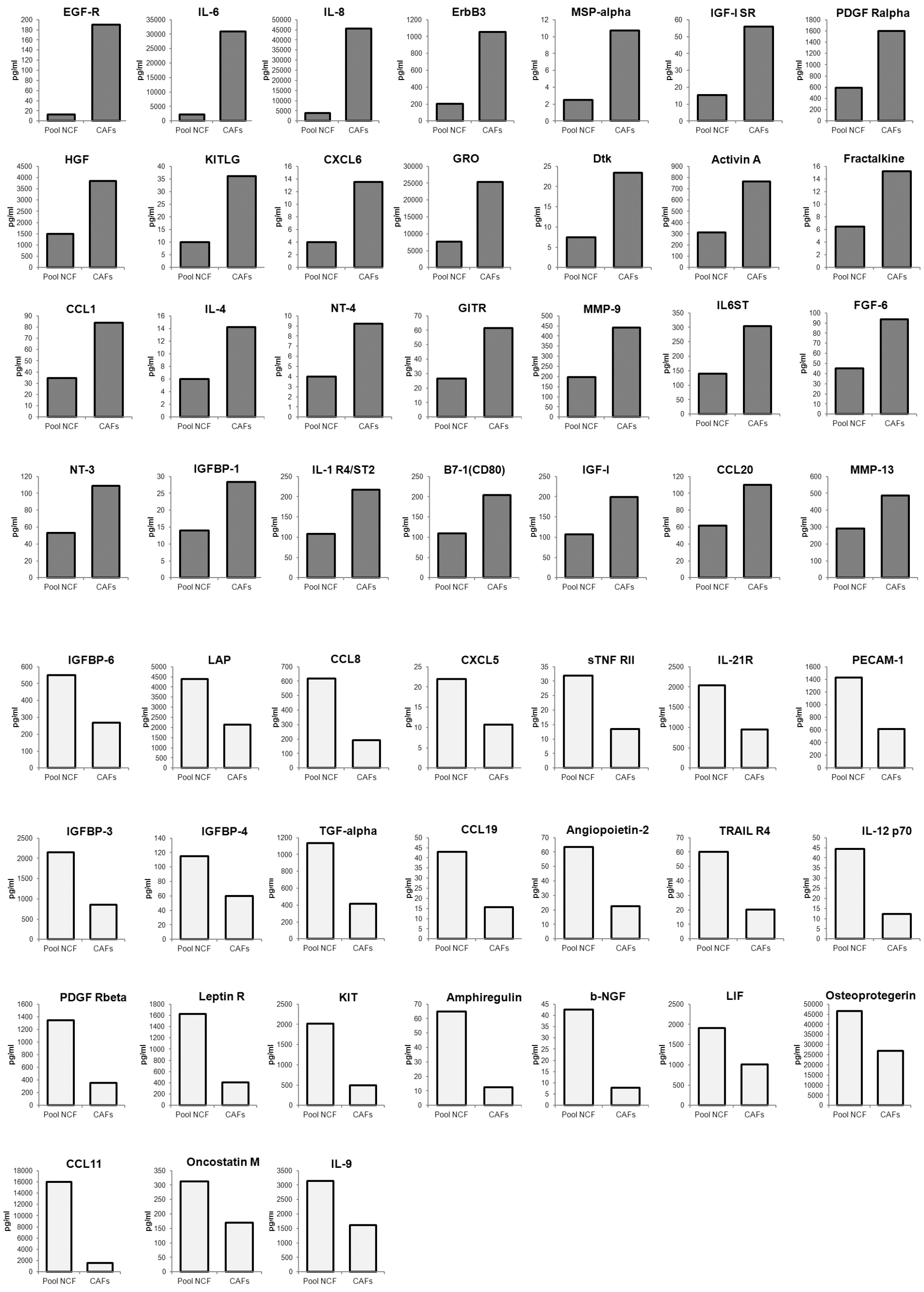

Supplementary Figure S5: Cytokine profile of a pool of three different normal colonic fibroblasts (NCF) conditioned medium compared to mean cytokine values of three different CAFs CM. The values were obtained using glass slide arrays from Raybiotech measuring 174 cytokines. Grey bars represent cytokines overexpressed in CAF vs NCF with at least 1.8 fold change. White bars are cytokines overexpressed in NCF vs CAF.

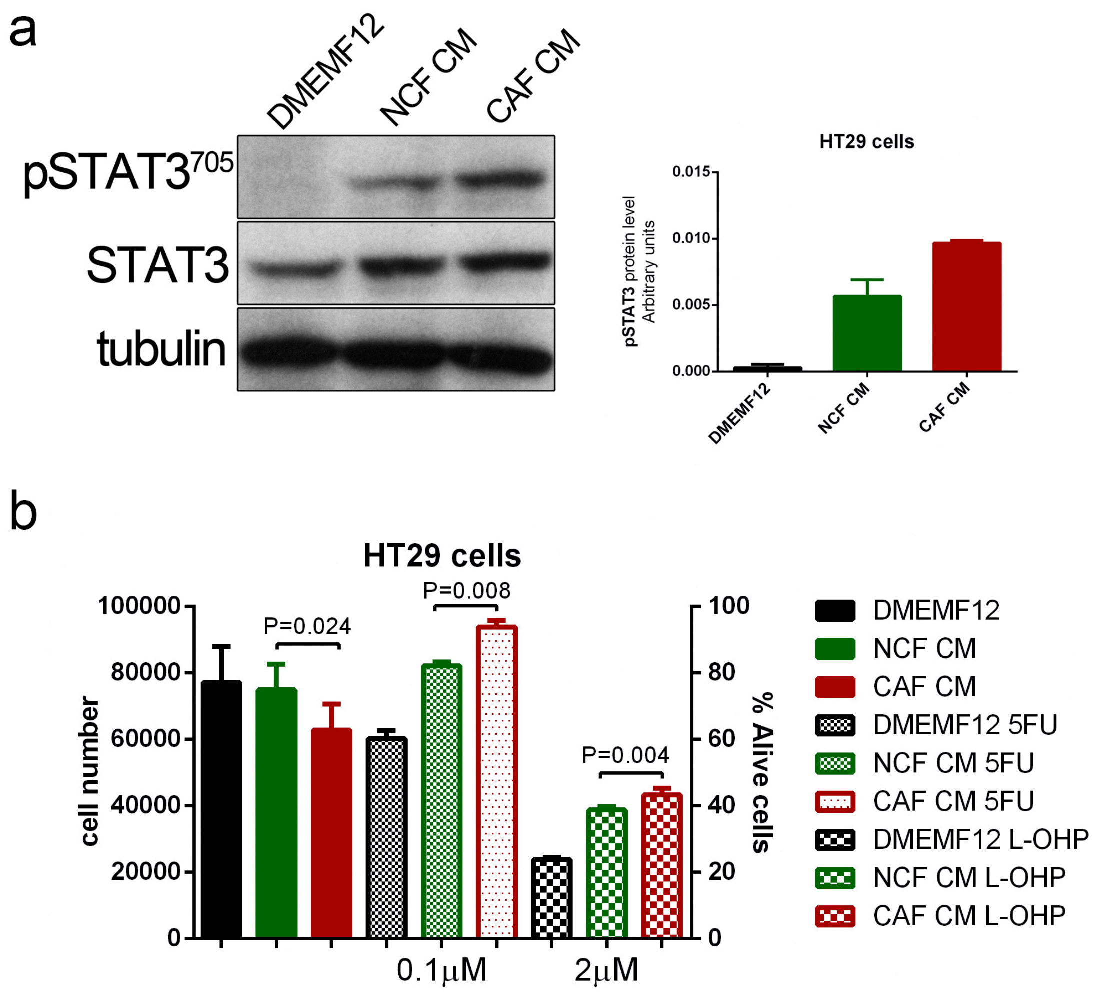

Supplementary Figure S6: a. Representative immunoblot (left panel) showing an hyperactivation of pSTAT3 in cells cultured in CAF-CM versus NCF-CM and mean values of three different independent experiments (right panel). **b.** Proliferation (left Y axis, solid color bars) and viability (right Y axis, dotted bars) of HT29 cells cultured with DMEMF12, NCF-CM and CAF-CM.

Supplementary Table S1: Flow cytometric analysis of cell cycle phases of HT29 and DLD-1 cells cultured with standard culture medium or CAF-CM

See Supplementary File 1
