# Supplementary material for: Carcinoma-associated fibroblasts affect sensitivity to oxaliplatin and 5FU in colorectal cancer cells
Source: Oncotarget. 2016 Aug 8;7(37):59766–80. doi: 10.18632/oncotarget.11121 (PMC5312347; doi:10.18632/oncotarget.11121)
Supplement: Supplementary file 2 [file oncotarget-07-59766-s002.docx]

**Supplementary table 1**. Flow cytometric analysis of cell cycle phases of HT29 and DLD-1 cells cultured with standard culture medium or CAF-CM.

|  |  | **Sub G0** | | | **G0/G1** | | | **S** | | | **G2/M** | | | **Aneuploidy** | | |
| --- | --- | --- | --- | --- | --- | --- | --- | --- | --- | --- | --- | --- | --- | --- | --- | --- |
|  |  | *48 hours* | *72 hours* | *96 hours* | *48 hours* | *72 hours* | *96 hours* | *48 hours* | *72 hours* | *96 hours* | *48 hours* | *72 hours* | *96 hours* | *48 hours* | *72 hours* | *96 hours* |
| **HT29 cells** | | | | | | | | | | | | | | | | |
| **No drug** | DMEMF12 | 0.6±0.1 | 1.7±0.9 | 1.3±0.3 | 44.5±1.4 | 42.4±0.6 | 43.1±0.8 | 17.5±1.9 | 18.4±0.4 | 19.2±3.4 | 24.4±1.3 | 29.3±0.9 | 28.7±1.6 | 12.5±1.9 | 7.4±1.6 | 7.5±1.5 |
|  | CAF-CM | 1±0.1 | 1.2±0.3 | 1.1±0.2 | **57.2±1.9** | **55.6±2.2** | **54±0.9** | 13.4±0.6 | 15.2±2.5 | 18.1±0.2 | **15.9±0.3** | **18.5±3.6** | 21.1±1.4 | 11.3±0.6 | 8.3±4.2 | 5.1±1 |
| **L-OHP** | DMEMF12 | 0.9±0.1 | 0.5±0.0 | 1.4±0.1 | 16.1±1.2 | 6.6±5.4 | 17±4.4 | 8.1±1.2 | 7.4±0.3 | 8.3±1.5 | 62.5±3.5 | 68.7±7 | 43.8±1.3 | 12.2±1.9 | 15.8±1 | 27±5 |
|  | CAF-CM | 1±0.2 | 1.3±0.2 | 1.7±0.0 | **22.8±1.1** | **20.4±1** | **25.3±0.9** | 12.4±1.8 | 11.2±1.5 | **17.9±0.2** | **49.6±1.2** | **46.1±0.5** | **29.1±0.7** | 11.5±0.4 | 19.7±0.2 | 23.4±0.4 |
| **5FU** | DMEMF12 | 0.7±0.2 | 2.8±0.3 | 2.7±0.6 | 37.7±1.2 | 23.2±3.9 | 27.5±4 | 32.7±1.5 | 40±1.3 | 41.2±1.9 | 17.1±0.5 | 25.7±5.3 | 18.2±2 | 9.7±0.9 | 5.9±0.1 | 9±0.1 |
|  | CAF-CM | 1±0.1 | 3±0.2 | 2.8±0.2 | **54.7±3.8** | 21.3±0.7 | 29.2±4 | **21.8±1.2** | **26.4±2** | **34.9±4.2** | 12.1±0.5 | **39.7±1** | 23±0.6 | 8.6±1.5 | 8±0.3 | 7.6±1.2 |
| **DLD-1 cells** | | | | | | | | | | | | | | | | |
| **No drug** | DMEMF12 | 2.9±0.3 | 3.1±0.5 | 4.6±2.6 | 9.9±1 | 4.7±0.6 | 23.5±1.1 | 4.2±0.4 | 3.2±0.2 | 4.5±0.8 | 27.2±0.9 | 17.9±2.7 | 26.3±3.8 | 55.6±1.2 | 71±2.7 | 40.9±0.8 |
|  | CAF-CM | 3±0.3 | 4.6±1.3 | 5.2±1.4 | **24.2±1.5** | **16.6±4.5** | **35.2±2.5** | 6.4±0.1 | 3.9±0.6 | 4.8±1.7 | 27.1±1.9 | 25.1±1.6 | 23.1±1.4 | **39.3±3.2** | **49.7±5.4** | **31.1±0.8** |
| **L-OHP** | DMEMF12 | 2.9±0.2 | 4±0.3 | 3.9±0.2 | 6.1±0.2 | 9.5±0.2 | 11.8±0.7 | 2.9±0.3 | 3.5±0.3 | 7.1±0.7 | 19.7±0.8 | 20.3±1 | 16.6±0.7 | 68.2±1.7 | 62.4±1.3 | 61.5±2.1 |
|  | CAF-CM | 3.5±0.1 | 4.9±0.2 | 5.7±0.0 | 9.2±1 | **18.7±0.4** | **18.4±0.4** | 4.9±2.1 | 3.9±0.7 | 6.29±0.0 | 21.5±0.7 | 21±1.4 | 22.7±0.3 | 62.1±0.1 | **46.6±0.7** | **46.1±0.0** |
| **5FU** | DMEMF12 | 2.8±0.7 | 2.5±0.1 | 3.3±1.5 | 9.9±0.8 | 7.5±1.6 | 13±0.9 | 4±0.4 | 3.2±0.4 | 6.6±2.2 | 27.4±1.2 | 19.7±1.6 | 21.5±1.9 | 56±0.3 | 67±3.6 | 55.7±3.4 |
|  | CAF-CM | 2.8±0.0 | 4.8±0.6 | 5.9±1.6 | **17.7±0.0** | **12.9±2.5** | **29.7±1.7** | 4.2±0.1 | 4±1 | 9±1.3 | 28.1±0.3 | 26.2±2.9 | 22±3.3 | **46±1.1** | **51.8±6** | **33.3±2.2** |

In bold, CAF-CM treated cells with statistically significant differences (P<0.05) compared with those cultured in DMEMF12 for the corresponding treatment/time.

Asynchronous HT29 and DLD-1 cells cultured in CAF-CM showed significantly more cells in G0/G1 than in those cultured in DMEMF12 for all the times assessed (48, 72 and 96 hours; P<0.05, except for HT29 cells after 72 hours and 96 hours of 5FU administration). This finding corroborates a slower proliferative ratio of cells in CAF-CM. Interestingly, after L-OHP addition, the percentages of HT29 cells in G2/M were significantly lower in CAF-CM than in standard medium (P<0.05), suggesting that some cells may complete cell division under the influence of soluble factors released by CAFs. In a similar way, after the addition of 5FU, CAF-soluble factors hinder the accumulation of cells in S-phase, allowing a relatively high percentage of cells to reach mitosis and divide. For DLD-1 cells under L-OHP treatment, no differences were seen in G2/M, but we did observe an accumulation of aneuploid cells that was lesser in CAF-CM. This fact, in combination with the higher percentage of cells in G0/G1 and the fewer necrotic cells observed in the flow cytometric analysis, suggests that some cells in CAF-CM overcome the mitotic slippage and divide normally, while most aneuploid cells probably die after defective mitosis in the next interphase. There was a similar outcome for 5FU, with very few cells arrested in S phase.
